# FISH Detection of *PML-RARA* Fusion in ins(15;17) Acute Promyelocytic Leukaemia Depends on Probe Size

**DOI:** 10.1155/2013/164501

**Published:** 2013-03-28

**Authors:** Lynda J. Campbell, Paul Oei, Ross Brookwell, Jake Shortt, Nicola Eaddy, Ashley Ng, Edward Chew, Peter Browett

**Affiliations:** ^1^Victorian Cancer Cytogenetics Service, St Vincent's Hospital Melbourne, Fitzroy, VIC 3065, Australia; ^2^Department of Medicine, St Vincent's Hospital, University of Melbourne, Fitzroy, VIC 3065, Australia; ^3^LabPLUS, Auckland City Hospital, Auckland 1023, New Zealand; ^4^Sullivan & Nicolaides Pathology, Indooroopilly, Brisbane, QLD 4068, Australia; ^5^Department of Haematology, Alfred Hospital, Prahran, VIC 3004, Australia; ^6^Department of Haematology, Auckland City Hospital, Auckland 1023, New Zealand; ^7^Department of Haematology, Royal Melbourne Hospital, Parkville, VIC 3050, Australia; ^8^Department of Molecular Medicine & Pathology, University of Auckland, Auckland 1142, New Zealand

## Abstract

The diagnosis of acute promyelocytic leukaemia (APL) is usually confirmed by cytogenetics showing the characteristic t(15;17), but a minority of patients have a masked *PML/RARA* fusion. We report ten patients with APL and no evidence of the t(15;17), in whom the insertion of *RARA* into *PML* could not be demonstrated by initial FISH studies using a standard dual fusion probe but was readily identified using smaller probes. Given the need for rapid diagnosis of APL, it is important to be aware of the false negative rate for large *PML/RARA* FISH probes in the setting of masked rearrangements.

## 1. Introduction

Acute promyelocytic leukaemia (APL) is characterised by the reciprocal 15;17 translocation involving the *PML* gene on 15q24, and *RARA* gene on 17q21 in more than 90% of cases. This translocation creates a *PML/RARA* fusion gene on the derivative chromosome 15 [1]. Occasional cases of complex translocations involve 15, 17 and other partner chromosomes, or insertions of 15 into 17 and vice versa, all resulting in a *PML/RARA* fusion [2]. There are also rare variant translocations involving *RARA* and other partner genes: *PLZF*, *NPM*, *NuMA*, *STAT5b, PRKAR1A, FIP1L1*, and *BCOR* [3–6]. In a series of APL cases without the standard t(15;17), most contained the *PML/RARA* fusion caused by an insertion and the fusions were usually demonstrated by both RT-PCR and fluorescence *in situ* hybridization (FISH) [7]. 

We present ten APL cases without cytogenetic evidence of t(15;17) in whom RT-PCR identified the PML/RARA fusion transcript, but initial FISH with standard probes showed no abnormality. Subsequent FISH revealed a small *PML/RARA* fusion signal in all cases on an apparently normal chromosome 15. Thus, all cases appeared to represent insertions of *RARA* into 15q24. Using the dual fusion probe from Abbott Molecular Inc., the *PML* signal swamped the tiny *RARA* signal. Careful examination showed a fusion signal in 3 cases, but, even in retrospect, there was no evidence of a fusion signal in 7 cases. Depending on which probe is used, a negative FISH result in APL does not, therefore, exclude the diagnosis.

## 2. Patients and Materials and Methods

Seven cases were identified from the records of 135 APL patients analysed by the Victorian Cancer Cytogenetics Service (VCCS) (cases 1, 3, 4, 5, 8, 9, and 10) over an 11-year period from 2002, two cases were identified from 25 APLs analysed by the Cytogenetics Department of LabPLUS, Auckland City Hospital (cases 2, 7) over a 5-year period from 2002 and one case was analysed by the Cytogenetics Department of Sullivan and Nicolaides Pathology, Brisbane (case 6) in 2005.

Cytogenetic studies were performed using standard protocols and FISH was performed according to the manufacturers' instructions. Five FISH probes were used: LSI *PML/RARA* dual colour translocation probe (Abbott Molecular Inc., Des Plaines, IL, USA), LSI *PML/RARA* dual colour dual fusion translocation probe (Abbott), LSI *RARA* dual colour break apart rearrangement probe (Abbott), *PML/RARA* translocation probe (extra signal) and *PML/RARA* translocation dual fusion probe (Cytocell Technologies, Cambridge, UK). 

FISH was performed on cytogenetic preparations fixed in 3 : 1 v : v methanol/glacial acetic acid, derived preferentially from short-term (usually less than 24 hour) cultures. Slide preparations were hybridized with the various locus-specific probes using codenaturation and overnight hybridization. Analysis was performed using a Zeiss Axioplan 2 Epifluorescence microscope and analysed by ISIS software (Metasystems, Altu*β*heim, Germany). A minimum of 200 cells were scored for each probe by two scorers. Cut-off values for false positive results (below which the result was regarded as normal) were <1% for the dual fusion probes, 3% for the RARA break apart probe, 10% for the Cytocell *PML/RARA* extra signal probe, and 10% for the *PML/RARA* single fusion probe. Karyotypes were described according to ISCN (2009) [8].

For quantitative t(15;17) *PML-RARA* gene analysis, RNA was purified using Trizol as per the manufacturer's instructions (Invitrogen), 1st-strand cDNA transcribed using SuperScript II (Invitrogen) and absolute quantitative PCR performed using Taqman assay on a Fast Real-Time ABI7500 PCR instrument (Applied Biosystems) using absolute quantitation standards.

## 3. Results and Discussion

APL is usually diagnosed on the bone marrow morphology and confirmed by the presence of the t(15;17) and detection of the PML/RARA fusion transcript via RT-PCR [3]. The t(15;17) is reported in 92% of APL cases, with 2% having simple or more complex variants, another 4% with insertions of *RARA* into *PML* or *PML* into *RARA,* and the rest with *RARA* fused to partner genes other than *PML* or, in 1%, with no RARA rearrangement [3]. We have identified ten cases of APL without a t(15;17) that appear to have produced a *PML/RARA* fusion gene by inserting a small segment of *RARA* into the *PML* gene on one cytogenetically normal chromosome 15. Seven of these cases were studied at the VCCS between 2002 and 2012, during which time 135 new cases of APL were diagnosed. Thus, the incidence of these cryptic insertions was 7/135 (5%), comparable to previously published series of cryptic abnormalities in APL [3, 9]. No specific morphological features distinguished this group as there were both classic hypergranular (*n* = 8) and variant hypogranular cases (*n* = 2). The RT-PCR results showed that there was no uniformity with regard to the PML breakpoint (Table 1), and immunophenotyping did not show any striking differences compared with the majority of APL cases (results not shown).

Clinical details of all patients are summarized in Table 1. The median age at diagnosis was 43 years (range 22–78 years) and there was an equal sex distribution. Survival data is available for 9/10 patients and 7/9 remain in complete remission 2–119 months after diagnosis. Two patients received modified Pethema protocols [11] and six were treated according to the Australian Leukaemia and Lymphoma Group studies APML3 [10] or APML4 [12]. All but two patients developed DIC and there were two early deaths—one of a presumed cerebral haemorrhage prior to the commencement of therapy (case 3) and one attributed to infection at 1 month post diagnosis (case 4).

The karyotypic, molecular, and FISH data are presented in Table 1. Seven of the ten patients had a normal karyotype; three contained additional abnormalities unrelated to the *PML-RARA* fusion. FISH in all cases using either the LSI *PML/RARA* t(15;17) dual colour dual fusion translocation probe (Abbott) in 9/10 cases or a single fusion translocation probe (Abbott) in one case failed to reveal a *PML-RARA* fusion signal despite RT-PCR identifying a PML/RARA transcript in all cases.

**Figure 1:**
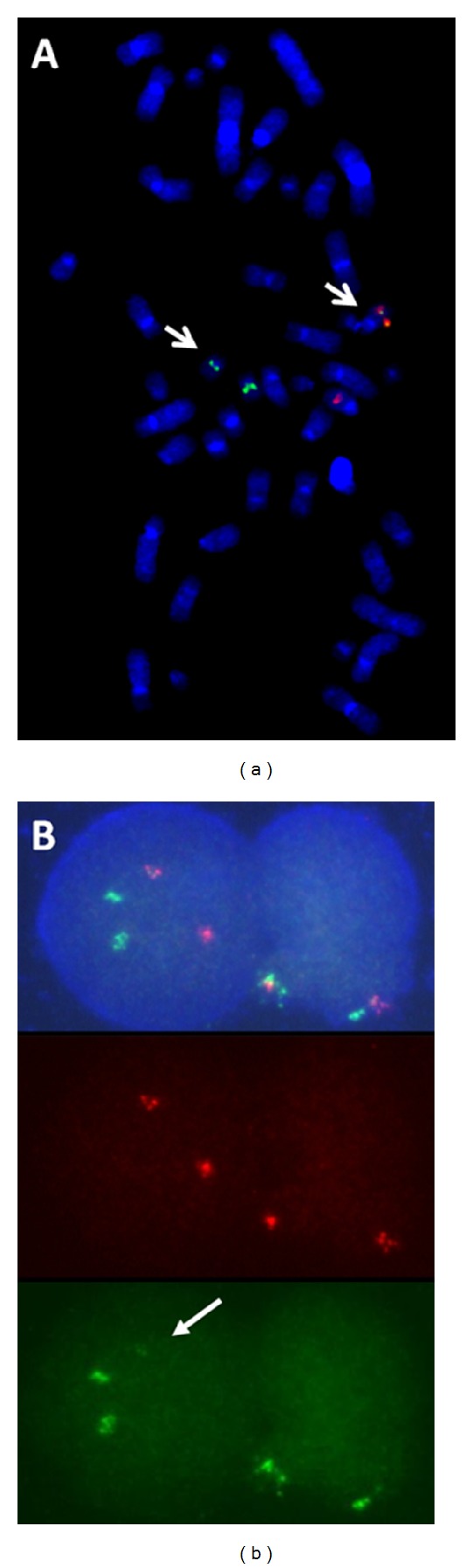
(a) Metaphase spread with the Cytocell *PML/RARA *extra signal probe showing a fusion signal on one chromosome 15 and a diminished *RARA* (green) signal on one chromosome 17. (b) Interphase cells with the Vysis dual colour dual fusion probe showing two red and two green signals in the top panel, two red signals in the middle panel, and two green signals plus a tiny third green signal (arrowed) in the bottom panel.

The Abbott dual fusion probe contains fluorescently labelled DNA that covers approximately 180 kb and 335 kb either side of the *PML* loci on chromosome 15 including all of the known BCR regions of *PML* and approximately 700 kb of chromosome 17 spanning all the breakpoint region of *RARA* (http://www.abbottmolecular.com/products/oncology/fish/hematology-probes.html). It was, therefore, puzzling that these probes were unable to detect a fusion signal in patients producing the PML/RARA transcript, whereas the Cytocell probes clearly revealed a fusion signal in all cases (Figure 1(a)). 

The major difference between the probes lies in the size of the respective probes. Both the *PML* and *RARA* segments of the extra signal probe from Cytocell are only approximately 40 kb in size, 100x smaller than their Abbott counterparts. The Cytocell *PML/RARA* translocation dual fusion probe is larger than the extra signal probe but the fluorescently labelled segments that span either side of the *PML* locus are only 151 kb and 174 kb and those spanning RARA are 167 kb and 164 kb in size. In three cases, a review of the Abbott dual fusion probe result revealed a tiny green (*RARA*) signal underlying one *PML *signal, only visualized using the single-colour Spectrum Green filter (Figure 1(b)). In the remaining 7 cases, despite careful examination, the extra *RARA* signal could not be seen. Apparently, the discrepancy between the size of the Abbott *PML* signal and very small *RARA* segment inserted into 15q24 allowed the *PML* signal intensity to quench the *RARA* signal, whereas the less disparate intensities of the two signals using the Cytocell probes allowed the fusion to be visualized.

Occasional cases of APL with normal cytogenetics and normal FISH studies have been reported previously [14, 15]. Indeed, Brockman et al., when reporting on the efficacy of the original dual fusion *PML/RARA* probe, noted the difficulty in identifying masked *PML/RARA* fusions. In their series of 38 APL cases, two did not initially show a fusion signal and it was only by observing a tiny additional RARA signal, via the single-pass SpectrumGreen filter, located in the same position as one *PML* signal that the *PML/RARA* fusion was revealed [15]. This is the first report to consistently identify these cryptic rearrangements using alternate FISH probes.

Given the importance of a rapid and reliable test to confirm the diagnosis of APL, it is critical that the possibility of a false negative result using standard FISH probes is considered and that alternate probes are available for rare cases of insertions resulting in the *PML/RARA* fusion.

## Figures and Tables

**Table 1:** 

Case no.	Age/sex	WBC × 10^9^/L	Dx	DIC	Karyotype	FISH probes*					PML- BCR^#^	Treatment protocol	Survival (months)
						SF (A)	DF (A)	BA (A)	ES (C)	DF (C)			
1	22/F	0.6	M3	No	46,XX	NT	−	NT	+	+	1	ALLG APML3 protocol [10]	2+
2	25/F	6.5	M3	Yes	46,XX	−	−/+	NT	+	NT	1	Modified Pethema protocol [11]	103+
3	30/M	161	M3V	Yes	46,XY,add(4)(q34), add(5)(q12)[4]/46,XY[17]	NT	−	NT	+	+	1	Nil	0
4	30/F	1.9	M3	Yes	46,XX,add(7)(q22)[10]/ 46,XX[20]	NT	−	−	+	+	1/2	ALLG APML4 protocol [12]	1
5	43/M	2.0	M3	Yes	46,XY	NT	−/+	−	+	+	3	ALLG APML3 protocol	119+
6	43/F	42	M3V	Yes	46,XX	NT	−	−	+	NT	3	NA	NA
7	50/F	11.5	M3	Yes	46,XX	−	−/+	NT	+	NT	3	Modified Pethema protocol	105+
8	57/M	1.1	M3	No	46,XY,t(2;13)(p25;q22) [18]/45,X,-Y[3]/46,XY[26]	NT	−	−	+	+	1	ALLG APML4 protocol	17+
9	59/M	6	M3	Yes	46,XY	NT	−	NT	+	+	1	ALLG APML4 protocol	41+
10	78/F	7.5	M3	Yes	46,XX	NT	−	NT	+	+	3	ALLG APLM3 protocol	55+

*FISH probes abbreviations: SF(A): single fusion probe—LSI PML/RARA dual colour translocation probe (Abbott Molecular Inc.); DF(A): dual fusion probe—LSI PML/RARA dual colour dual fusion translocation probe (Abbott); BA(A): break apart probe—LSI RARA dual colour break apart rearrangement probe (Abbott); ES(C): extra signal probe—PML/RARA translocation probe (Cytocell); DF(C): dual fusion probe—PML/RARA translocation dual colour probe (Cytocell); NT: not tested; NA: not available; −: not visible; −/+: only visible via single band-pass filters; +: observed; ^#^PML/RARA RT-PCR identification of the variant transcripts: 1 refers to the *PML* bcr1 within intron 6 and 2 to the *PML* bcr2 with variable breakpoints within exon 6 and 3 to *PML* bcr3 within intron 3 [13].
